# Tea compound-saliva interactions and their correlations with sweet aftertaste

**DOI:** 10.1038/s41538-022-00123-9

**Published:** 2022-02-09

**Authors:** Pik Han Chong, Jianshe Chen, Danting Yin, Lanxi Qin

**Affiliations:** 1 Lab of Food Oral Processing, School of Food Science and Biotechnology, Zhejiang Gongshang University, Hangzhou, Zhejiang 310018 China; 2 Firmenich Aromatics (China) Co., Ltd., No. 3901, Jindu Road, Minhang District, 201108 Shanghai China

**Keywords:** Biochemistry, Engineering

## Abstract

Huigan is an important sensory attribute which is commonly used as a quality indicator evaluation of tea products. Previous studies showed a strong correlation between the lubrication behavior of saliva-tea compound mixture and the sensory perception of Huigan from trained panelists. This work was further designed to investigate how the effect of tea consumption on the rate of saliva secretion and its functional properties including total protein content of saliva (TPC), salivary α-amylase (AMY) and lipase activity (LP). A quartz crystal microbalance with dissipation monitoring (QCM-D) was applied to reveal the adsorption behavior of human whole saliva and how the salivary film is affected by the presence of tea compounds. Results showed a significant positive correlation among TPC, LP and Huigan intensity for subjects who are Huigan-sensitive. Compared to the desorption of salivary film, the desorption of saliva-EC/EGC (epicatechin/epigallocatechin) mixture from the gold surface by QCM-D observation showed a significant effect on Huigan intensity in sensitive group when comparing to the salivary layer (blank).

## Introduction

Tea is one of the most popular beverages generally consumed around the world. The flavor of tea has been extensively studied in the last few decades. It is commonly known that tea flavor can be affected by many factors including plant varieties, climates, place of origin, processing methods, the types and amount of tea compounds presented in the tea leaves^1^. For green tea alone, as many as 31 flavor attributes (i.e. beany, green herb-like, burnt, nutty, floral, fruity, bitter, astringent, mint, fermented etc.) and aftertaste have been developed (Lee and Chambers^2^) for flavor description^2^. In China, a typical example of aftertaste is the sweet aftertaste, a sensation of sweet taste lasting in the mouth and throat after the consumption of tea. The sweet aftertaste of tea has not gained much attention and the underlying mechanism remains unclear. Our previous studies have demonstrated that the sweet aftertaste of tea can lead to an elevated salivation for an extended period of time and a term of Huigan, (a Chinese term for sweet aftertaste) was specifically used for this sensory attribute^3^. L-theanine contributes the umami and sweet taste to tea^4^; trilobatin is a unique compound found in sweet tea leaves (*Lithocarpus polystachyus* Rehd.)^5^; epicatechins (EC) and epigallocatechins (EGC) were reported to contribute sweet aftertaste to the green tea^6^. Due to the sweetness characteristic of those tea compounds, the Huigan intensity of L-theanine, trilobatin, epicatechins and epigallocatechins were studied and found to correlate well with the lubrication behavior of saliva-tea compound mixture. According to sensory studies, the highest Huigan intensity rated among the studied tea compounds was the mixture of EC and EGC solution. To follow previous observation and to understand the underpinning mechanisms of Huigan, this work extends previous study^3^ to investigate the interactions between tea compounds and human saliva and their impacts on the sensory perception of tea.

It is generally agreed that human saliva plays an important role in the food oral processing and sensory perception^7^. This is because saliva works as a media for the transport of tastants from the food to the taste receptors and an essential agent for bolus formation for swallowing. The oral lubrication behavior of saliva is altered when it is mixed with food and/or food components. The interaction of salivary components with food compounds impacts on and alters flavor perception^8^. This makes it important to understand the interaction of human saliva with food (and components) which could affect salivary properties (e.g., salivary flow rate, total protein content and enzyme activity after food consumption) as well as sensory properties such as aftertastes.

Saliva is mainly produced by submandibular, parotid and sublingual glands. It comprises of about 98% water and 2% other substances such as salivary proteins, electrolytes, enzymes, antimicrobial compounds^9,10^. There is about 1 to 2 mg/ml of proteins present in the saliva that composes of enzymes, glycoproteins, immunoglobulins and peptides such as proline-rich proteins, statherin, cystatins and histatins. The common inorganic substances found in saliva are potassium, sodium, bicarbonate and chloride^11^. Enzymes in saliva take part in the sensory perception during food oral processing. For example, amylase influences the sensory attributes (e.g., creaminess, stickiness, roughness, and etc.) of a starch-based food; lingual lipase could be a factor affecting the oral perception of dietary fat^8,12^, salivary esterases influence ester release during wine consumption^13^. Saliva can even function as an effective emulsifier to disperse oil/fat during an eating process, possibly due to the presence of surface-active biopolymers in the saliva^14^.

Quartz crystal microbalance (QCM) is a high resolution mass sensing technique that measures the mass in nano scale by acoustic waves. The mass change on the quartz crystal surface is obtained by detecting the change of the frequency that oscillates the crystal. Extensive researches have been conducted in literature to study the solution-surface interface at molecular level by QCM technique^15^. Instead of measuring the frequency itself, quartz crystal microbalance with dissipation monitoring (QCM-D) can also determine the amount of energy dissipation from the system by shutting off the driving voltage to the crystal. The viscoelastic properties (shear viscosity and elastic modulus) of the adsorbed film can then be extracted by recording the frequency and dissipation at multiple harmonics of resonant frequency^16^. Surface properties of human saliva have been studied using QCM-D technique^17–21^. In this work, the QCM-D technique was applied to investigate surface adsorption of human saliva in the presence of tea compounds. Previous study has confirmed possible interactions between tea compounds and saliva and their influences on Huigan perception. The objectives of the study were to further investigate such interactions using QCM technique, and, on the other hand, to reveal whether tea consumption affects saliva secretion and certain saliva compositions. The findings from this work could help to interpret the oral physiological implications of Huigan sensation of tea consumption.

## Results and discussion

### Huigan intensity

Table 1 summarizes the sensory intensity of Huigan for various tea compound solutions (bottled water was served as control), following the procedure described in previous studies^3^. The Huigan intensity of the mixture of EC and EGC rated by sensitive group was the highest and showed significantly different compared to other samples in both the oral and oral-pharyngeal stage (*p* < 0.05). However, nonsensitive group did not perceive the Huigan much differences among the samples, particularly the intensity rated in the oral-pharyngeal stage (*p* > 0.05).

**Table 1:** The Huigan intensity of the tea compound solutions rated by sensitive and nonsensitive group in the oral and oral-pharyngeal stage.

Sample	Sensitive Group		Nonsensitive Group	
	Mean ± 95% CI (Oral stage)	Mean ± 95% CI (Oral-pharyngeal stage)	Mean ± 95% CI (Oral stage)	Mean ± 95% CI (Oral-pharyngeal stage)
Water	2.8 ± 0.7^b^	3.1 ± 0.7^b^	2.6 ± 0.6^b^	2.5 ± 0.5^a^
L-theanine	2.4 ± 0.8^b^	2.4 ± 0.8^b^	2.7 ± 0.6^ab^	3.1 ± 0.6^a^
Trilobatin	3.4 ± 0.9^b^	2.9 ± 0.6^b^	2.8 ± 0.8^ab^	3.1 ± 0.8^a^
EC/EGC	5.2 ± 1.3^a^	5.2 ± 1.0^a^	4.1 ± 1.0^a^	3.9 ± 1.0^a^

Each sensory group consisted of 6 subjects and the experiment was conducted in triplicate. Different letters in the same column indicate significant difference from other samples. Results are adopted from Chong et al.^3^.

Flow rate, total protein concentration, α-amylase and lipase activity of human whole saliva

The salivary flow rate (SFR) has been found highly individual dependent. Aframian et al. and others^22–24^ observed the highest value of 2.87 ml/min for unstimulated saliva (US) flow, but once stimulated, the SFR becomes much higher, ranging between 0.05 and 7.0 ml/min. The SFR of US in our study ranged from 0.17 to 1.05 ml/min, towards a lower side of the literature range. Very interestingly, a different saliva flow pattern was observed between the Huigan-sensitive group and Huigan nonsensitive group. The SFR of US on average from the sensitive group was found to be 0.36 ml/min, significantly lesser than that from the nonsensitive group (0.53 ml/min), with a *p* value of 0.001. What we can make from this difference is still open for discussion, but it seems clear that oral physiological conditions play an important role in influencing one’s Huigan sensation. For subjects who already have high saliva flow, a further increase of saliva secretion may have limited influence on the sensation of mouth watering and therefore gives very little impact on Huigan perception.

**Fig. 1:**
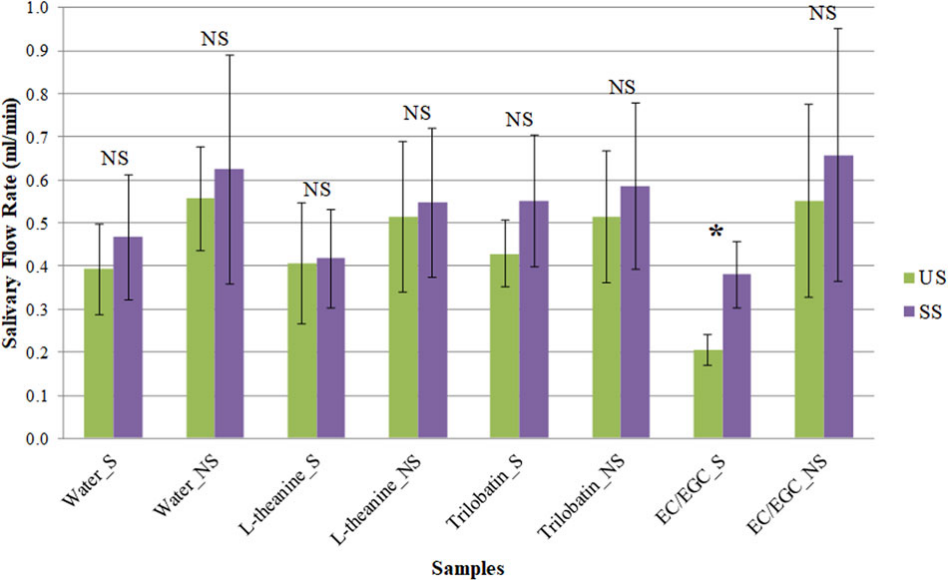
The change of salivary flow rate as affected by the consumption of samples in sensitive and nonsensitive group. (US and SS are the unstimulated and stimulated saliva before and after the consumption of sample, respectively; S and NS denote Sensitive and Nonsensitive group, respectively. * and NS indicate significant and not significant difference within each sample of the particular sensory group, respectively. The error bars represent 95% confidence interval).

Figure 1 illustrates the change of SFR as influenced by the consumption of sample for both studied groups. In general, the consumption of each sample increased SFR in both groups, although no significant difference (*p* > 0.05) was noticed except the consumption of EC/EGC in the sensitive group (*p* < 0.05). It is worth mentioning that the SFR from most of the subjects (10 out of 12) increased after consuming EC/EGC, suggesting that consuming the mixture of EC and EGC solution would positively elevate the salivary secretion.

**Fig. 2:**
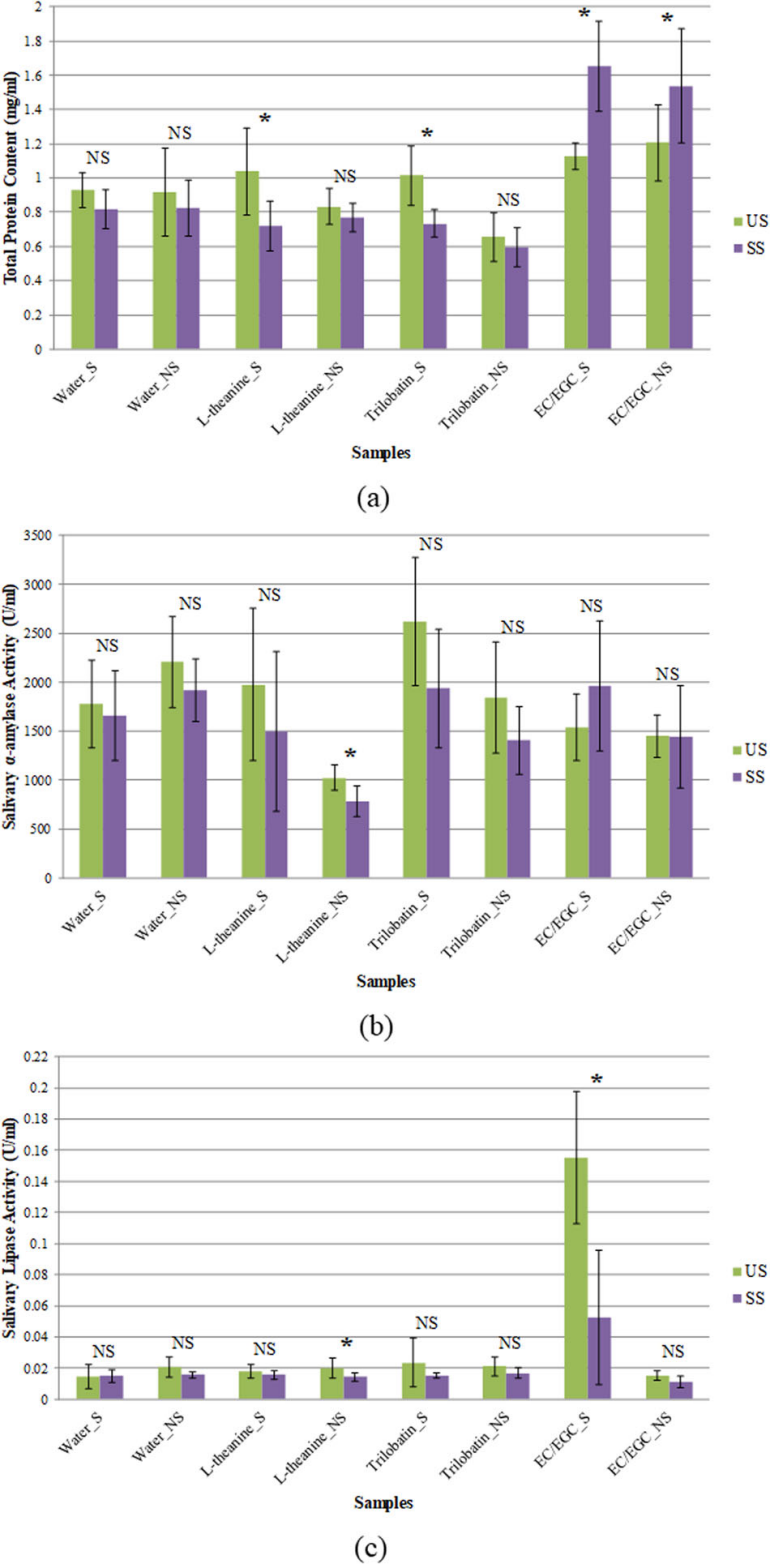
The change of salivary component as affected by the consumption of samples by sensitive and non-sensitive group. **a**–**c** The total protein content of saliva, salivary α-amylase, and lipase activity, respectively. US and SS are the unstimulated and stimulated saliva before and after the consumption of sample, respectively; S and NS denote sensitive and non-sensitive group, respectively. An asterisk symbol and NS indicate significant and not significant difference within each sample of the particular sensory group, respectively. The error bars represent 95% confidence interval.

The change of saliva composition after tea consumption seems to be more complicated. The TPC of US from the studied subjects ranged from 0.18 to 1.92 mg/ml (data not shown). Figure 2 shows the change of TPC, salivary AMY and LP after consuming the sample for both groups. Not surprising, the influence of sample consumption on the studied parameters also varied (increased, decreased or little changed) among individuals as well as samples. Figure 2a shows that the TPC of saliva was decreased significantly when consuming L-theanine and trilobatin in sensitive group, while the consumption of EC/EGC increased protein secretion significantly in both groups (*p* < 0.05). If observed individually, the TPC of saliva from 11 subjects was increased after drinking the mixture of EC and EGC solution. Results from this study suggested that consumption of EC/EGC tends to increase salivary protein secretion, probably for better protection of oral surfaces, because tannins are harmful for oral structures due to the complex formation with histatin and basic proline-rich protein to produce insoluble compounds^25–27^. The friction coefficient of the saliva-EC/EGC mixture was higher than other samples in previous studied, revealing that the EC and EGC reduced the lubrication properties caused by the interaction of the salivary protein with EC/EGC^3^.

The consumption of the sample generally reduced the salivary AMY in both groups except the sample EC/EGC in the sensitive group (Fig. 2b). A significant difference was found (*p* < 0.05) on salivary AMY after consuming L-theanine for nonsensitive group. A reduction of salivary LP activity was also noticed after drinking the sample in both groups (Fig. 2c). The enzyme activity of salivary LP was relatively low in comparison with salivary AMY. LP was significantly reduced (*p* < 0.05) after the consumption of L-theanine in nonsensitive group and EC/EGC in the sensitive group. When linking the results of SFR to TPC and enzyme activity, an increase of SFR caused by the consumption of tea compound samples reduced the TPC (except EC/EGC), salivary AMY and LP. The real cause behind these observations is not clear at this stage, but we speculate that the excess amount of saliva secreted could be mainly protein-free fluid which leads to a diluting effect to TPC, AMY, and LP.

**Fig. 3:**
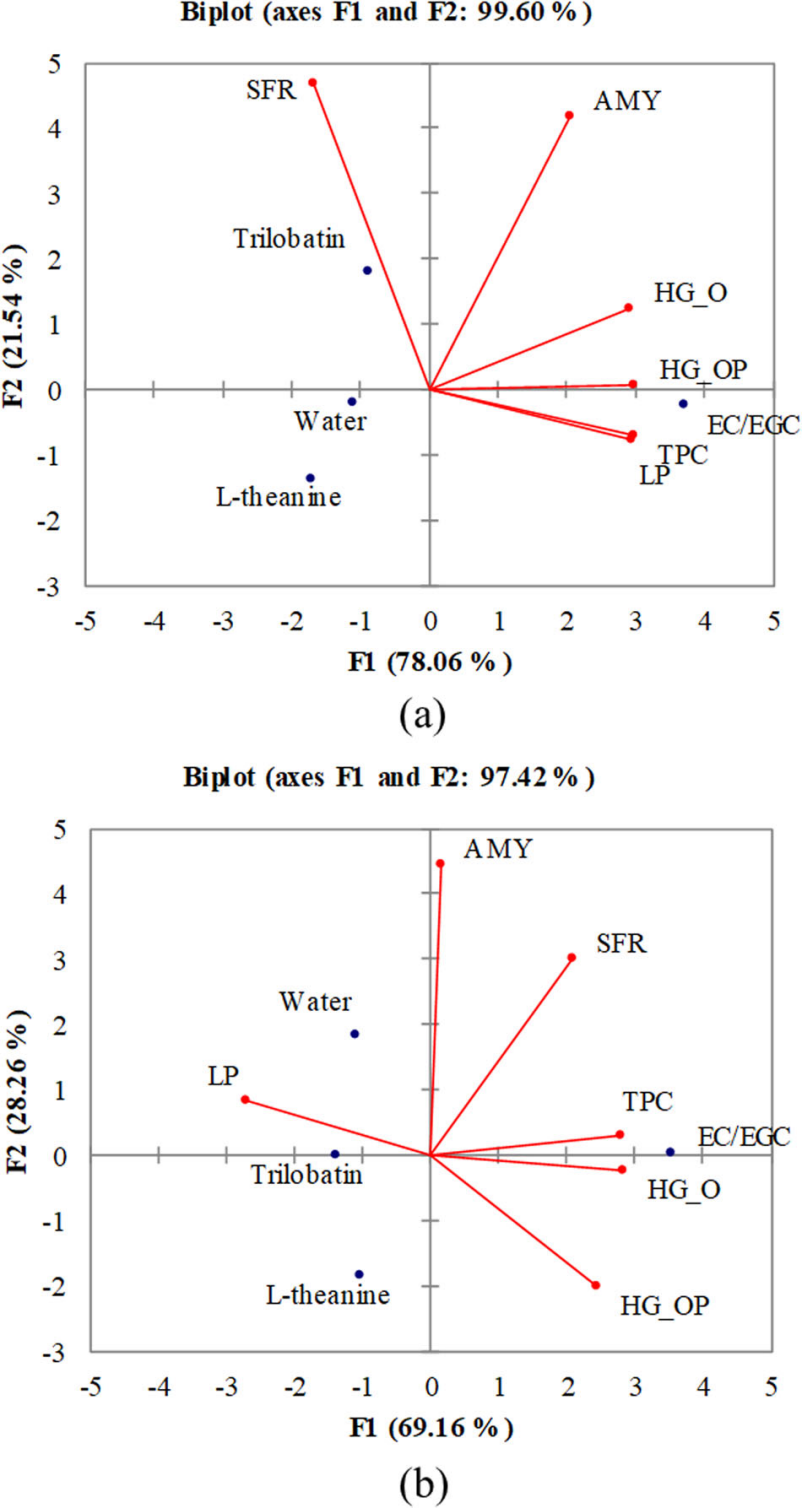
Principal component analysis of human saliva after the consumption of the sample. **a**, **b** The sensitive and non-sensitive group, respectively. The salivary flow rate, total protein content, α-amylase activity, lipase activity, Huigan intensity rated in oral stage and Huigan intensity rated in oral-pharyngeal stage are denoted as SFR, TPC, AMY, LP, HG_O, and HG_OP, respectively.

PCA was applied for the analysis of Huigan intensity, SFR, TPC of saliva, salivary AMY and LP after consuming the sample. Results are illustrated in Fig. 3. There was 99.60 and 97.42% of the initial variability of the data attributed by Factor 1 (F1) and Factor 2 (F2) in the sensitive group and nonsensitive group, respectively. Table 2 summarizes the factor loading of the analytical variables. A good fit of the variables with the factor solution is indicated by a high factor loading. The factor loadings of F3 in both groups were relatively low (<0.60) and were dropped from the analysis. It was shown in the sensitive group that the TPC of saliva, salivary AMY and LP, and Huigan intensity rated in oral (HG_O) and oral-pharyngeal (HG_OP) stage associated mostly with F1 and SFR for F2. It is clearly observable in the Fig. 3a that variables HG_OP, LP and TPC are highly correlated. The positive and significant correlation between TPC and LP, TPC and HG_OP, as well as LP and HG_OP was further verified by Pearson’s correlation analysis, with a *p* value of 0.006, 0.012, and 0.027, respectively. It is also clear from the figure that EC/EGC stands out from all other samples and is closely related to the studied variables (HG_OP, LP and TPC). This may imply that EC/EGC is the key compounds for Huigan sensation and positively affected the TPC of saliva and salivary LP. Even though it is great to see high percentage in PCA analysis for both sensitive and nonsensitive groups, one should be aware that the number of samples involved in the analysis was relatively small. It is reasonable to expect that the percentage will become much lower should more samples were included in the study.

**Table 2:** Factor loadings of the analytical variables.

Variables	Sensitive Group		Nonsensitive group	
	F1	F2	F1	F2
SFR	−0.567	0.823	0.736	0.675
TPC	0.992	−0.124	0.984	0.068
AMY	0.680	0.732	0.057	0.997
LP	0.989	−0.132	−0.958	0.187
HG_O	0.976	0.216	0.991	−0.055
HG_OP	0.993	0.011	0.858	−0.451

*SFR* Salivary Flow Rate, *TPC* Total Protein Content, *AMY* α-amylase Activity, *LP* Lipase Activity, *HG_O* Huigan rated in oral stage, *HG_OP* Huigan rated in oral-pharyngeal stage.

The important factors that associated positively with F1 in the nonsensitive group were SFR, TPC, HG_O, and HG_OP, negatively for LP, while F2 positively correlated with AMY (Table 2). Based on Fig. 3b the TPC was positively associated with HG_O and negatively with LP. In addition to the distribution of the samples, the EC/EGC was located near to TPC and HG_O, indicating that sample EC/EGC was also the key tea compounds, which may even have the sensory impact to nonsensitive group. Pearson’s correlation revealed that the TPC was positively and significantly correlated with HG_O (*p* = 0.049) but negatively correlated with LP (*p* = 0.034). It seems that salivary LP after consuming the sample was the main factor to discriminate sensitive (positively correlated with TPC and HG) and nonsensitive group (negatively correlated with TPC and HG).

### Tea compound and saliva interactions based on quartz crystal microbalance with dissipation

**Fig. 4:**
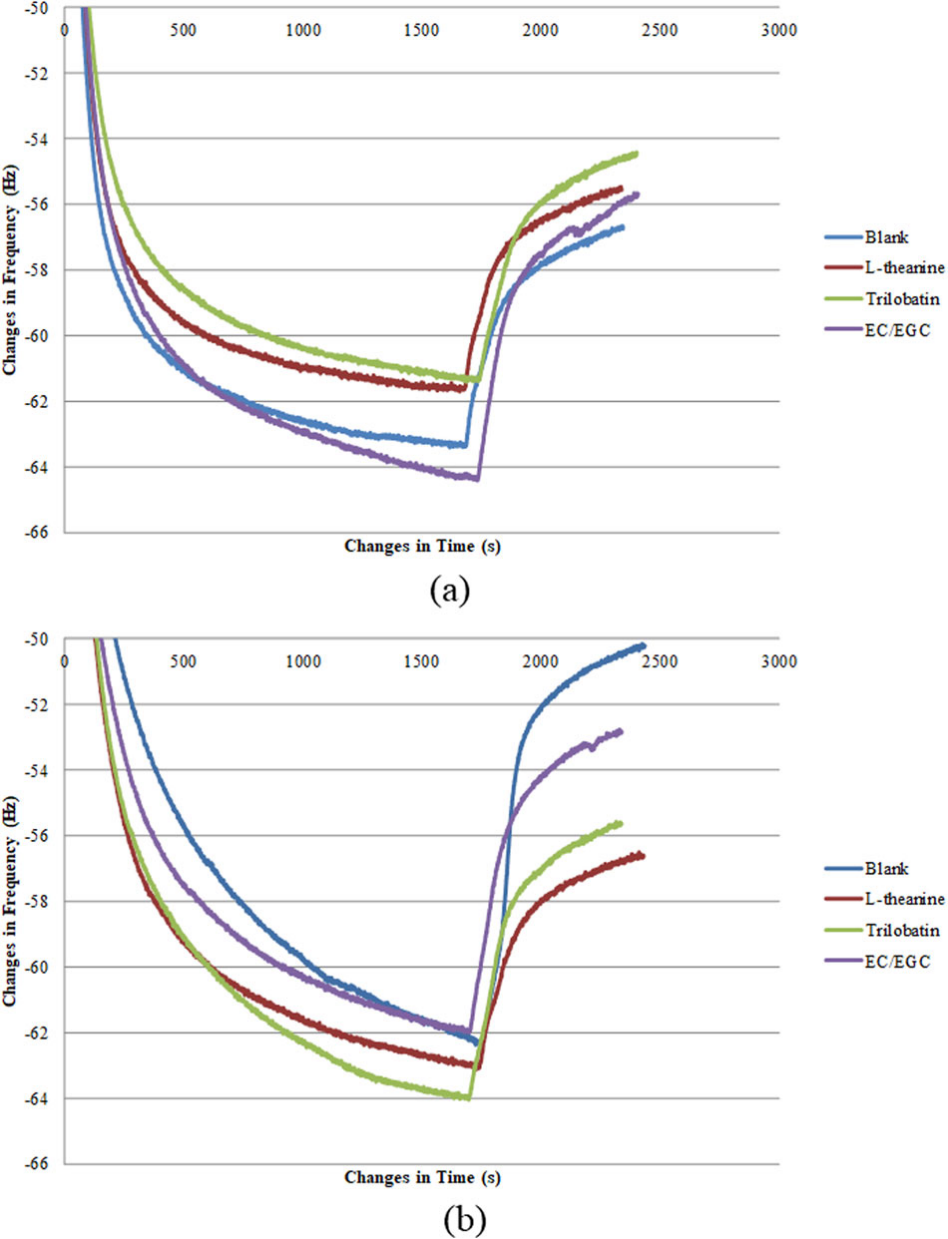
Representative curves for the frequency change over time for the 3rd overtone generated by the adsorption of saliva-sample mixture onto the gold sensor. **a**, **b** Sensitive group and non-sensitive group, respectively.

**Fig. 5:**
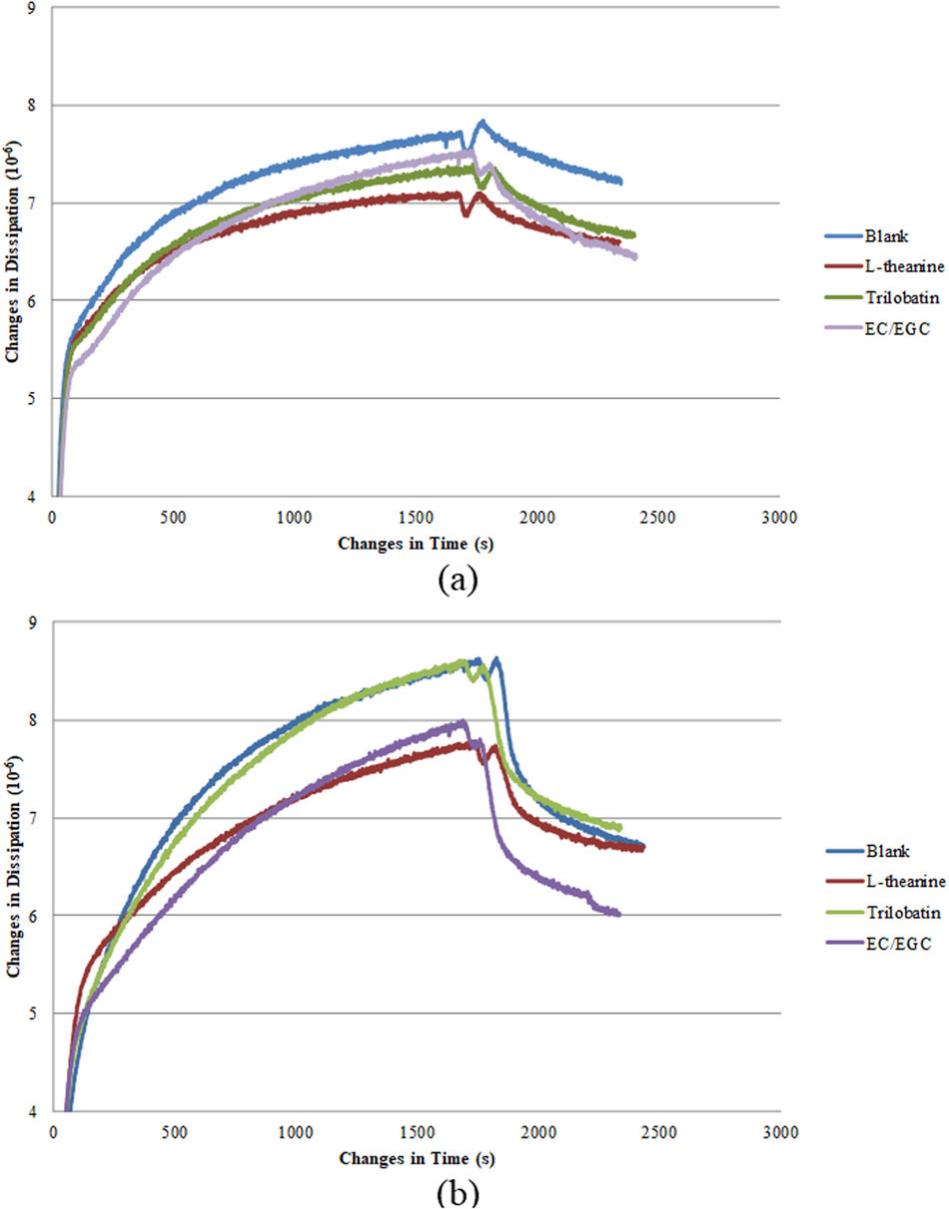
Representative curves for the dissipation change over time for the 3rd overtone generated by the adsorption of saliva-sample mixture onto the gold sensor. **a**, **b** Sensitive group and non-sensitive group, respectively.

The formation of the saliva-sample film on the gold surface was studied by applying QCM-D. The changes in frequency and dissipation (3^rd^ overtone) resulted from the adsorption of saliva-sample mixture onto the gold surface followed by a buffer rinse for stabilizing the layer are illustrated in Figs. 4 and 5. According to QCM theory, a lower frequency indicated a higher mass adsorption at the gold surface, and the mass uptake was accompanied by a rise in dissipation. There was initially a sharp decrease in frequency change and increase in dissipation change when the saliva-sample mixture was introduced to the sensor surface. This was followed by a gradual and close steady state at a later stage, inferring a high adsorption rate in the beginning, followed by a gradual decrease in adsorption rate. The sodium phosphate buffer was then delivered to the sensor surface in the chamber for a final rinsing step. This was accompanied by a sudden increase in frequency change and decrease in dissipation change, implying a possible desorption at the surface. In Table 3, QCM results were further analyzed for both sensitive and nonsensitive groups. Four QCM parameters relating to frequency and dissipation were presented for four sample system. Marks “a” and “b” were used to indicate whether statistical difference exists. Results showed that while frequency showed significant differences, there was no significant difference between sample systems, with the exception of EC/EGC sample system. The frequency and dissipation of saliva (blank) under steady state were −62.33 Hz and 7.46 ppm for the sensitive group, −61.63 Hz and 8.48 ppm for nonsensitive group. It was noticed in the Fig. 4 a, b and Table 3 that a layer with higher mass formed by saliva-EC/EGC mixture in sensitive group and saliva-trilobatin/L-theanine mixture in the nonsensitive group. When comparing the dissipation change between sensitive and nonsensitive group during the adsorption of saliva-sample onto the gold surface (Fig. 5a, b), a faster increase in dissipation found in nonsensitive group, revealing that a softer film of saliva-sample mixture was formed^18,20^. Furthermore, higher dissipation of saliva-sample film (shown in Table 3) was found in nonsensitive group than in sensitive group, which confirmed a softer film of saliva-sample formed in nonsensitive group. It should be taken note that the dosage of tea compounds in the QCM study was conformed to the sensory study. However, the changes of frequency and dissipation among samples within each sensory group were not significant due to the low concentration of tea compound solutions used in the study. The low amount of tea compounds might have limited the interaction with saliva. The interaction between saliva and the tea compounds with varied concentrations had to be further investigated.

**Table 3:** The frequency and dissipation (3^rd^ overtone) of saliva-sample film from sensitive and nonsensitive group.

Sample	*f*_1_ (Hz) Mean ± 95% CI	*f*_2_ (Hz) Mean ± 95% CI	*D*_1_ (ppm) Mean ± 95% CI	*D*_2_ (ppm) Mean ± 95% CI
Sensitive group				
Blank	−62.33 ± 2.11^b^	−56.16 ± 1.18^a^	7.46 ± 0.45^a^	6.86 ± 0.65^a^
L-theanine	−61.46 ± 0.24^b^	−55.45 ± 0.24^a^	6.82 ± 0.47^a^	6.46 ± 0.27^a^
Trilobatin	−63.11 ± 3.52^a^	−56.06 ± 3.21^a^	7.62 ± 0.62^a^	6.95 ± 0.56^a^
EC/EGC	−64.94 ± 1.32^b^	−55.52 ± 0.33^a^	7.62 ± 0.24^a^	6.17 ± 0.58^b^
Nonsensitive group				
Blank	−61.63 ± 0.92^b^	−50.87 ± 1.3^a^	8.48 ± 0.15^a^	6.75 ± 0.10^b^
L-theanine	−63.21 ± 0.51^b^	−55.81 ± 1.66^a^	8.06 ± 0.59^a^	6.70 ± 0.03^b^
Trilobatin	−63.00 ± 1.46^b^	−55.71 ± 0.10^a^	8.03 ± 0.99^a^	6.46 ± 0.87^a^
EC/EGC	−61.61 ± 0.61^b^	−52.39 ± 0.90^a^	8.32 ± 0.70^a^	6.32 ± 0.58^b^

*f*_1_ = the final frequency when saliva-sample film was steadily adsorbed to the gold sensor, *f*_2_ = the final frequency after a buffer rinse, *D*_1_ = the final dissipation when saliva-sample film was steadily adsorbed to the gold sensor, *D*_2_ = the final dissipation after a buffer rinse.

Different letters indicate significant difference between f_1_ and f_2_ and between D_1_ and D_2_ of the particular sample in the particular sensory group.

**Fig. 6:**
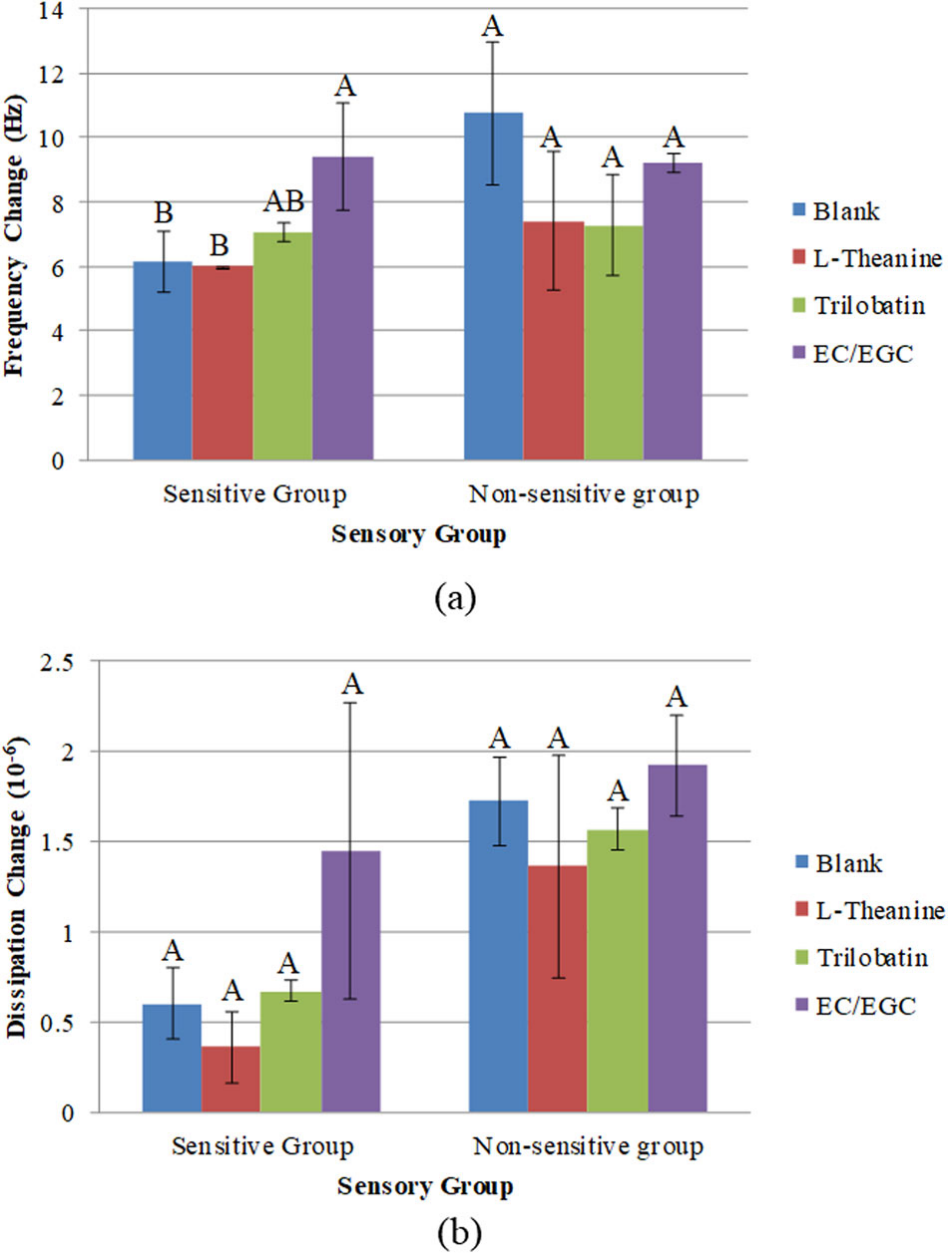
The change of frequency and dissipation after stabilization of the saliva-sample layer by a buffer rinse. **a**, **b** Frequency and dissipation, respectively. Means without the same letter indicate significant difference in the particular sensory group. The error bars represent 95% confidence interval.

The *t* test on frequency and dissipation after stabilizing the saliva-sample film by a buffer rinse was analyzed (see Table 3). There was a significant increase in frequency (*p* < 0.05) after stabilization of the film on all the samples in both the sensitive and nonsensitive group. In addition, the dissipation was not significantly reduced (*p* < 0.05) among the sample except saliva-EC/EGC in the sensitive group; for the sensitive group, a significant decrease (*p* > 0.05) of dissipation was observed for all the samples except saliva-trilobatin. In the present study, the saliva-EC/EGC complexes formed a steady layer from the sensitive group in comparison with nonsensitive group. The change of frequency and dissipation during buffer rinse of the saliva-sample film is further explained and presented in Fig. 6. It was noted in the Fig. 6a that the frequency change of saliva-EC/EGC (9.4 Hz) film was significantly higher than salivary film (6.2 Hz) (*p* < 0.05) in the sensitive group, which explained that the complex of saliva-EC/EGC film removed from the gold sensor surface was more than the salivary film itself (blank). A few of studies had been done to investigate the interaction between salivary protein and tannins^28–30^. The interaction of certain salivary proteins with tannins caused a precipitation by forming large aggregates. These aggregates might have been formed before, or during the time when they were delivered to the gold sensor surface. Based on the previous work^3^, the sensory study showed that the highest Huigan intensity was the EC/EGC mixture and high friction coefficient of the mixture with saliva was observed at the sliding speed lower than 0.5 mm/s from the sensitive group in the tribological study. Those findings indicated that the EC/EGC mixture did not just contribute to astringency (increase of friction in the oral cavity) and Huigan sensation while reacting with saliva, but also revealed by QCM-D study. The underlying mechanisms need to be further studied to explain the correlations among them. However, no significant differences were found (*p* > 0.05) on the frequency change among the samples in nonsensitive group. In addition to the dissipation change shown in Fig. 6b, no significant differences were noticed among the samples in both groups. Yet the change of dissipation observed from nonsensitive group was seemed to be higher than the sensitive group among all the samples, indicating that the viscoelastic properties of the saliva-sample layer formed from the sensitive group was more stable after a buffer rinse compared to nonsensitive group.

In conclusion, this work investigated the salivary secretion and certain salivary biochemistry as influenced by the consumption of certain tea compounds and the correlations between these properties and sensory perception of Huigan intensity. SFR and TPC of saliva from both groups show a positive effect after the consumption of the EC-EGC mixture giving the highest Huigan-tasting. The consumption of water, L-theanine and trilobatin somewhat reduces the TPC, salivary AMY and LP, attributed by the increased secretion of protein-free watery saliva. PCA studies the correlations among SFR, TPC, AMY, LP, HG_O, and HG_OP stage after the consumption of studied samples. Results from the sensitive group indicate a strong positive correlation among TPC, LP, and HG_OP after consuming samples, with the EC/EGC being the main tea compounds affecting them. For nonsensitive group, the TPC is positively associated with HG_O but negatively with LP after the consumption of samples, and the EC/EGC is the key compound reflecting the TPC and HG_O. The study of other compositions of saliva such as ions and other types of proteins (e.g., proline-rich proteins) are required to establish a more ideal correlation between Huigan sensation and those salivary parameters.

QCM-D studies show that the interaction between saliva of the sensitive group and EC/EGC forms a high mass of complex rigid layer on the gold sensor surface. When comparing a salivary film (blank) and saliva-EC/EGC film during buffer rinse, the latter showed more loss of mass from the gold sensor surface. The viscosity and elasticity of the saliva-tea compound complexes could be further investigated to understand the influence of tea compounds on salivary film. Overall, the results in the current work showed the differences between sensitive and nonsensitive groups. Findings suggest that tea consumption has a certain impact on saliva secretion and salivary biochemistry and the interactions between saliva and tea compounds could be a molecular basis of Huigan sensation of tea products.

## Methods

### Materials

L-theanine trilobatin, epicatechins and epigallocatechins were purchased from Wuhan Yuancheng Gongchuang Technology Co., Ltd. Nongfu Spring bottled drinking water was used as control in the study except QCM-D measurement. The total protein assay kit and α-amylase assay kit were purchased from Nanjing Jiancheng Bioengineering Institute Jiangsu, China. The commercial lipase was obtained from Aspergillus Niger lipase, Sigma-Aldrich, St. Louis, MO, USA. Chemicals of 4-methylumbelliferyl 7-oleate, sodium taurodeoxycholate and sodium azide were purchased from Sigma-Aldrich (Shanghai) Trading Co., Ltd. Ethylenediaminetetra-acetic acid, dithiothreitol and Aspergillus Niger lipase were obtained from Xilong Chemical Co., Ltd, Jiangsu Qiangsheng Function Chemistry Co., Ltd and Sigma-Aldrich, St. Louis Mo, USA, respectively. Tris-HCl and CaCl_2_ were purchased from Beyotime Institute of Biotechnology (Shanghai) Co., Ltd. Phenylmethylsulfonyl fluoride was obtained from Beijing Solarbio Science and Techonology Co., Ltd. For QCM-D measurement, ethanol (Xilong Scientific, China), sodium phosphate dibasic heptahydrate (Shanghai Macklin Biochemical Co., Ltd, China), ammonium hydroxide and hydrogen peroxide (Shanghai Aladdin Biochemical Technology, Co., Ltd, China), 11-Mercaptoundecanoic acid (11-MUA), N-(3-dimethylaminopropyl)-N’-ethylcarbodiimide hydrochloride (EDC), N-hydroxysuccinimide (NHS) and sodium dihydrogen phosphate anhydrous (Aladdin Industrial Corporation, China) were all used as received.

### Sensory analysis

Sensory training and subject evaluation of Huigan intensity were described in our previous work^3^. Briefly, the panelists attended a sensory training to understand the general attributes of tea (e.g., Huigan, sweetness, bitterness, sourness, astringency etc.). Furthermore, they were trained to rate the Huigan intensity by using different concentration of the tea brew. The capability of panelists for scaling Huigan intensity was assessed in the validation study by evaluating a few of tea brew samples. The results from the training showed that Huigan was perceived on both the tongue and throat, thus it could be an integrated sensation perceived from the oral cavity and throat. For the preparation of tea compound solutions, the concentration for EC/EGC, trilobatin, L-theanine were 400/100, 30, and 200 ppm, respectively. The panelists were asked to drink 15 gram of sample which was served at room temperature. The drinking procedure was accomplished by directing the sample into the mouth for 5 s and rating the Huigan intensity 10 s after swallowing it. The Huigan intensity was rated in two approaches based on 11-point scale: oral stage, denoted as O (without swallow) and oral-pharyngeal stage, denoted as OP (with swallow). Due to the individuals’ variation in response to Huigan perception, the 12 trained panelists were equally divided into 2 groups according to their sensitivity of Huigan perception based on the sensory results: sensitive group (S) who perceived Huigan correctly and nonsensitive group (NS) who were not sensitive to Huigan sensation of tea brew or tea compound solutions^28^.

### Saliva collection

A consent form was signed by the panelists for the participation of saliva collection and sensory evaluation study. Ethical permission was approved by University Ethics Committee (Ref no. 2016120124). The saliva collection was performed at 10 am of the day. Subjects were asked to refrain from drinking and eating 1 h before the experiment. For the analyses of total protein content (TPC), α-amylase activity (AMY) and lipase activity (LP) of saliva, the unstimulated saliva (US) was passively accumulated and expectorated to a cup over a period of 5 min after the mouth was properly rinsed. The panelists were asked to rest for 5 min before consuming the sample. A 15 ml of sample was then delivered into the mouth and swallowed 5 s later. The saliva stimulated by the sample was immediately collected for another 5 min right after swallowing. In order to observe the adsorption/desorption behavior of tea compound solution on salivary film, the stimulated saliva was collected by continuously chewing a piece of parafilm (Bemis, USA) for 5 min for the QCM-D measurement. Collected saliva was then pooled together by group type and centrifuged at 14000 × *g* at 4 °C for 30 min. The supernatants were collected and stored at −80 °C until being analyzed.

### Salivary flow rate

The salivary flow rate (SFR) was recorded for each subject before and after consuming the sample. Assuming 1 ml of saliva equals to 1 gram, the SFR (ml/min) was calculated by dividing the total volume of collected saliva, *V* (ml), by the collection time, *t* (min).

### Total protein content, α-amylase activity and lipase activity of saliva determination

The TPC was measured according to bicinchoninic acid (BCA) method using a total protein assay kit. Bovine serum albumin (BSA) was used as standard and the results were expressed in concentration (mg/ml). The salivary AMY was determined using the iodine-starch method with α-amylase assay kit. For a known concentration of the substrate, a blue complex is produced by adding iodine solution to the unhydrolyzed starch. The enzymatic activity was expressed in activity units (U), in which 1 unit means the hydrolysis of 1 mg starch per mL of saliva during 30 min of reaction time at 37 °C. The LP of saliva was determined by fluorimetry as described by Méjean et al.^31^. The LP of saliva was compared against that of the commercial lipase. The intensity of sample fluorescence was determined using a multi-mode microreader with the excitation filter at 355 nm and emission filter at 460 nm (Synergy H1, BioTek Instruments, Inc., Winooski, VT, USA) after a reaction of 30 min at 37 °C^29^.

### Quartz crystal microbalance with dissipation

The cleaning procedure of the quartz crystal surface was performed by following the recommended procedures provided by Biolin Scientific, the instrument manufacturer. The gold-coated sensor (Biolin Scientific, Sweden) with a fundamental frequency of 5 MHz was cleaned in a UV/Ozone chamber (HWOTECH, China) for 10 min. A 5:1:1 mixture of ultrapure water (Merck Millipore, US), 25% ammonia and 30% hydrogen peroxide was heated up to approximately 75 °C. The sensor was placed in the heated solution for 5 min, followed by rinsing it with ultrapure water and dried with nitrogen gas. The sensor was then placed in the UV/Ozone chamber for 10 min. The binding of salivary protein was accomplished by the surface activation of the gold sensor, as described by Yao et al.^21^, with slight modifications. After the cleaning process, the gold sensor was immersed in 10 mM 11-MUA (11-mercaptoundecanoic acid) in absolute ethanol solution at 60 °C for 24 h. The remaining 11-MUA was rinsed off with absolute ethanol, and the quartz crystal surfaces were dried with nitrogen gas. Before QCM-D measurements, a 1:1 (v/v) mixture of 100 mg/ml EDC and 100 mg/ml NHS in ultrapure water were prepared to activate the 11-MUA-coated quartz crystal surfaces for 1 h. The sensor was then rinsed with deionized water and dried with nitrogen gas.

The measurements were performed using Q-sense Explorer System Equipment (Biolin Scientific, Sweden). The activated gold sensor was placed in the chamber with its temperature controlled at 28 °C to mimic the oral surface temperature. It was initially filled with sodium phosphate buffer, pH 7, for 15 min to establish a stable baseline, followed by a mixture of 25% stimulated saliva and sample dissolved in the buffer for 40 min. The chamber was then stabilized by a buffer rinse for 15 min. The flow of the liquid was controlled at a flow rate of 0.1 ml/min.

### Statistical analysis

Data analysis was performed using XLSTAT. *T*-test was applied to compare the significant difference between unstimulated saliva and stimulated saliva caused by sample consumption; Analysis of Variance (ANOVA) was carried out to indicate the significant difference among the saliva-sample mixture in QCM-D studies. The Principal Component Analysis (PCA) on Huigan intensity, SFR, TPC of saliva, salivary AMY and LP after the consumption of tea compound sample (water as control) were analyzed. Pearson’s correlation was applied to study the correlation between Huigan intensity rated in both stages and frequency/dissipation change.

## Data Availability

The authors declare that the data support the findings of the study and are available from the corresponding author upon reasonable request.
